# Reliability study for the Rib Index in chest radiographs of a control group

**DOI:** 10.1186/1748-7161-10-S2-S9

**Published:** 2015-02-11

**Authors:** Konstantinos C Soultanis, Konstantinos Tsiavos, Theodoros B Grivas, Nikolaos A Stavropoulos, Vasileios I Sakellariou, Andreas F Mavrogenis, Panayiotis J Papagelopoulos

**Affiliations:** 1 1st Department of Orthopaedics, University of Athens, Medical School, University General Hospital “Attikon”, Chaidari, Athens 12462, Greece; 2 Department of Orthopaedics and Traumatology, “Tzaneio” General Hospital of Piraeus, Tzani and Afendouli 1, Piraeus 18536, Greece

## Abstract

**Background:**

The Rib Index, (RI), extracted from the double rib contour sign (DRCS) on lateral spinal radiographs to evaluate rib hump deformity, (RHD), in idiopathic scoliosis, (IS), patients, has been previously introduced. Although various papers using the RI have been published, no study on its reproducibility has been reported. The aim of this report is to estimate the variations of the RI in a number of a pair set of lateral chest radiographs (LCRs). The hypothesis was that the RI should have minimal variability for each subject having successive LCRs.

**Methods:**

Seventy randomized patients who were treated in the hospital for lung diseases (mainly pneumonia or other communicable lung diseases), were initially included in the study. Each of these patients had two successive LCRs (named A and B group of radiographs) at the radiological department of the hospital, by the same technician, during the course of their treatment. The radiation source - patient distance was constant. LCRs obtained at an incorrect patient’s position, or from patients who underwent a thoracic intervention and all LCRs with symmetric hemi-thoraces were excluded from the study. The LCRs of 49 patients were deemed suitable for inclusion in the study. The RI was calculated in both (A and B) LCRs of each patient. The statistical analysis included the following techniques: paired t-test, Pearson correlation coefficient and intra- and inter-observer error using the formula (SD/√2)/2, where SD is this of the differences of the two sets of measurement (As-Bs). The SPSS v16 statistical package was used.

**Results:**

In the 49 pairs of LCRs there was no statistical difference of the RI, (paired t-test p< 0.314). The RI in the A and B group of LCRs was perfectly correlated (correlation coefficient = 0,924, p < 0.0001). The intra-observer error was 0.0080 while the inter-observer error 0.0213 in terms of 95% CI.

**Conclusion:**

The RI proves to be a reliable method to evaluate the thoracic deformity and the effect of surgical or non-operative treatment on the IS RHD. RI is a simple method, a safe reproducible way to assess the RHD based on lateral radiographs, without the need for any further special radiographs and exposure to additional radiation.

## Background

All lateral spinal or chest radiographs in IS show a DRCS of the thoracic cage, a radiographic expression of the rib hump, (RH). The outline of the one hemi- thorax (convex) overlies the contour of the other hemi-thorax (concave) [1-3]. The RI, extracted from the DRCS on lateral spinal radiographs to evaluate the RHD in IS patients has been earlier introduced. This index evaluates the RHD in IS patients, attempting to create a safe and reproducible method to assess this deformity based on lateral radiographs [1-3].

Although this method has been used in a number of publications, no reliability study has been presented so far. The aim of this report is to estimate the variations of the RI in a number of a pair set of LCRs of patients. The hypothesis is that the RI should have minimal variability for each patient having successive LCRs.

The present study will attempt to answer to the following questions:

1. Has the RI the same value in successive radiographs of the same individual?

2. Does the same examiner measure the same value of RI in successive measurements in the same radiograph?

3. Does a second examiner measure the same value of RI in the same radiograph of the patient?

## Methods and materials

### Patients

Seventy randomized patients who were admitted to the medical department of the hospital for lung diseases (mainly pneumonia or other communicable lung diseases) during the years 2009-2013, were initially included in the study. Forty-nine of the seventy patients were deemed suitable for assessment. The exclusion criteria were LCRs obtained in an incorrect patient’s position, those of patients who underwent a thoracic intervention and all LCRs with symmetric hemi-thoraces without a DRCS. Forty one were males and twenty nine were females while their mean age was 58 years, (range 25 to 80 years). Each of them had two successive LCRs to assess the course of their treatment. In order to access the data of the patients this study was approved by the Ethical & Scientific Committee for Clinical Research at "ATTIKON" Hospital. The details of the included patients in the study are presented in the additional file 1. The institutional review board (IRB) permission was obtained.

### Measurements

**Figure1:**
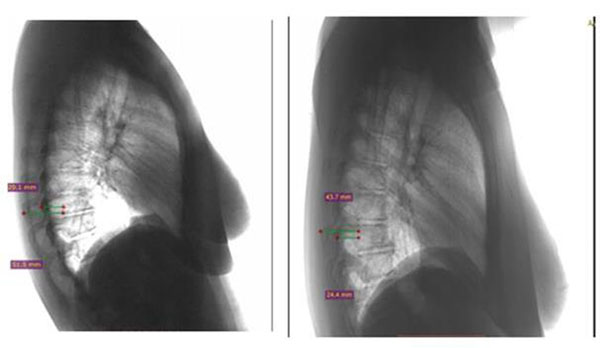
**Examples of two LCRs (group A and group B respectively).** Group A included the 1^st^ LCR and group B included the 2^nd^ LCR.

Two successive LCRs were obtained in a standardized way for each patient included in the study. The LCRs were divided into two groups, namely group A and group B. Group A included the 1^st^ LCR and group B included the 2^nd^ LCR as shown in Figure 1. The minimum interval time between the two successive LCRs was two days. The RI was calculated in this set (A and B) of LCRs of each patient. It should also be noted that the radiation source-patient distance remained constant and that the radiographs were performed by the same technician. Radiant Dicom Viewer software was the tool for the measurements on LCRs.

**Figure 2:**
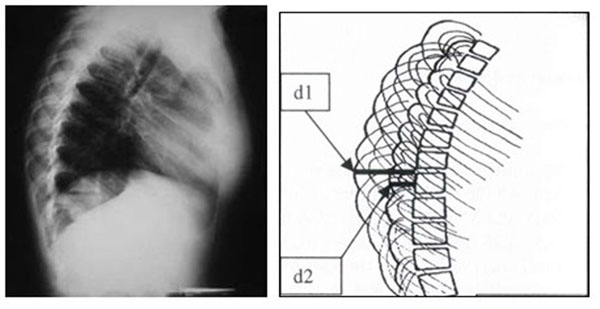
**The description of the RI method**. RI is calculated by the ratio of the two distances d1/d2 in LCRs where d1 is the distance between the most extended point of the most extending rib contour and the posterior margin of the corresponding vertebra on the LCRs, while d2 is the distance from the least projected rib contour and the posterior margin of the same vertebra.

The RI method (RI=d1/d2) is described in Figure 2.

### Statistical analysis

The statistical techniques used included: paired t-test in the set of A and B group of LCRs, correlation coefficient, intra- and inter-observer error in terms of 95% Confidence Interval, using the formula (SD/√2)/2, where SD is this of the differences of the two sets of measurement (As-Bs). The IBM SPSS v16 statistical package was used for the statistical analysis.

## Results

The results of the comparison of the RI of both groups are shown in Table 1. The paired t-tests are presented in order to assess whether there was a significant difference of the RI between the two groups (group A and group B). In Table 1 it is shown that in the 49 pairs of LCRs there was no statistical difference of the RI, p<0.314.

**Table 1:** Rib Index values in the 2 Groups (Group A and B).

**GROUPS**	**MEAN**	**N**	**Std. Deviation**	**Std. Error Mean**
A	1.663	49	0,2597	0,0371
B	1.6673	49	0,25250	0,03607

The mean values, standard deviation and standard error mean of the RI in the two groups (p<0.314)

The correlation between the values of the RI for both groups was calculated. Table 2 shows that the RI in group A and in group B was strongly correlated (r= 0.924 and p<0.000), therefore there was a high degree of agreement in the values of the RI.

**Table 2:** Coefficient Correlation. The Pearson correlation coefficient between the values of the RI in the two groups was statistical significant.

**Groups**	**N**	**Correlation coefficient**	**Sig.**
A and B	49	0.924	0,000

Table 3 shows the inter- and Table 4 the intra-observer error. The first and the second observer calculated twice the RI in ten days interval. The intra-observer error was 0.0080, while the inter-observer error 0.0213 in terms of 95% CI.

**Table 3:** Ιntra–observer error. This table shows the two successive measurements of RI by the same observer and its differences. The intra-observer error in terms of 95% CI was calculated using the formula (SD/√2)/2, where SD is this of the differences. The intra–observer error was 0.0080.

**RI 1^st^ measurement**	**RI 2^nd^ measurement**	**Difference**
1.33	1.33	0
1.41	1.42	0.01
1.41	1.44	0.03
1.79	1.78	0.01
1.84	1.81	0.03
1.95	1.95	0
1.72	1.71	0.01
1.3	1.34	0.04
1.71	1.75	0.04
1.66	1.67	0.01

**Table 4:** Inter–observer error. This table shows the two successive measurements of RI by the two observers and its differences. The inter-observer error in terms of 95% CI was calculated using the formula (SD/√2)/2, where SD is this of the differences between the two observers. The inter-observer error was 0.0213.

**RI :1^st^ observer’s measurement**	**RI: 2^nd^ observer’s measurement**	**Difference**
1.33	1.34	0.01
1.41	1.41	0
1.41	1.45	0.04
1.79	1.79	0
1.84	1.81	0.03
1.95	1.95	0
1.72	1.72	0
1.3	1.33	0.03
1.71	1.73	0.02
1.66	1.68	0.02

## Discussion

One of the principal advantages of RI is that it is simple, since no special radiograph is necessary [3] because only two LCRs, (the preoperative and the postoperative in case of IS patients surgically treated) are required. Yet the RI method provides the possibility for retrospective studies.

Adolescent scoliosis is a three-dimensional deformity [4]. Quite a few techniques are employed in order to assess the deformity of the spine and the thoracic cage [5]. The most commonly used is the Cobb angle method [6]. The most frequently used techniques for the evaluation of the vertebral rotation are these of Nash-Moe [7] and the Perdriolle [8-10]. These methods do not assess the degree of rib deformity affecting the thoracic cage [11]. The RI is considered an excellent method to assess RHD due to its simplicity and to the ability to be calculated on the lateral scoliosis film with no need for special imaging or additional exposure to radiation [11]. As it has been earlier noted the RI method extracted from the DRCS was introduced to evaluate the RHD in IS patients in an attempt to create a safe and reproducible method to assess the thoracic deformity based on lateral radiographs [11]. This assessment is actually the transverse plane thoracic deformity.

Therefore the answers to the above questions are:

### Question 1: Has the RI the same value in different radiographs of the same individual?

The results of this study show that there was no significant difference of the RI between the groups (group A and group B) and that it was strongly correlated. Therefore, it can be stated that the value of the RI is the same for each patient in successive radiographs.

### Question 2: Does the same examiner measure the same value of RI in different measurements of the same radiograph?

The findings suggest that the measurements, which were done twice, produced the same values of the RI for the same radiograph.

### Question 3: Does a second examiner measure the same value of RI of the same patient’s radiograph?

Based on the reliability study results presented in Table 4, the difference in the measurements of the two observers was insignificant in terms of 95% CI. Consequently, it can be claimed that the value of the RI of each patient’s lateral radiograph was the same.

## Conclusion

The RI method proves to be a reliable method to evaluate the thoracic deformity or the effect of surgical or conservative treatment on the IS rib-cage deformity (RH). Furthermore, it is a simple method and a safe reproducible way to assess the RHD based on lateral radiographs, without the need for further special radiographs and exposure to additional radiation.

This is the extended abstract of IRSSD 2014 program book [12].

## Consent

Patients were informed prior medical services or admission to the University Hospital "ATTIKON" that imaging and other medical data could be used in teaching and academic purposes.

## Competing interests

There are no competing interests to disclose.

## Authors' contributions

KCS: drafted the text and searched the literature. TBG: Conceived the idea of RI, drafted the text and searched the literature. NAS: drafted the text, searched the literature and implemented the statistical analysis. KT: Implemented the measurements on LCRs and drafted the text. All authors contributed their professional skills to the inclusions of the text. All authors have read and approved the final manuscript.

## Supplementary Material

Additional file 1 Click here for file

## Declarations

Publication charges for this collection were funded by IRSSD 2014 Sapporo.

This article has been published as part of *Scoliosis* Volume 10 Supplement 2, 2015: Research into Spinal Deformities 9: Short Papers from the IRSSD 2014 Meeting. The full contents of the supplement are available online at http://www.scoliosisjournal.com/supplements/10/S2.
