# Supplementary material for: Reliability study for the Rib Index in chest radiographs of a control group
Source: Scoliosis. 2015 Feb 11;10(Suppl 2):S9. doi: 10.1186/1748-7161-10-S2-S9 (PMC4331769; doi:10.1186/1748-7161-10-S2-S9)
Supplement: Additional file 1 [file 1748-7161-10-S2-S9-S1.rtf]

Patient
Number	Date of 1st
Measurement	Date of 2nd
Measurement	D1 1st Meas.	D2 1st Meas.	RI 1st Meas.	D1 2nd
Meas.	D2 2nd
Meas.	RI 2nd Meas.	Excluded Patients	
1	16/11/2013	31/10/2013	37.2	19.2	1.93	45.7	23.6	1.93		
2	28/02/2012	06/03/2012	51.2	36.6	1.4	54.8	32.8	1.67		
3	04/05/2012	13/05/2012							thoracic intervation	
4	08/11/2011	03/11/2011	48	25.3	1.89	55.5	34.4	1.61		
5	29/03/2011	09/01/2013	47	31.8	1.47	52.3	33.1	1.57		
6	24/04/2012	19/04/2012	35.1	24.6	1.42	35.1	24.6	1.42		
7	07/01/2012	08/12/2010	44	31.2	1.41	43.4	28.7	1.51		
8	21/09/2011	09/02/2011	56.1	34.3	1.63	52.5	32.6	1.61		
9	04/08/2012	04/11/2010	42.4	35.9	1.18	56.8	32.4	1.75	incorrect patient's position	
10	28/11/2011	25/10/2011	42.3	22.5	1.88	41	22.5	1.82		
11	19/07/2009	14/01/2010	49.6	27.8	1.78	50.9	29.1	1.74		
12	12/02/2011	15/02/2011	48.2	27.8	1.73	47.6	27.4	1.73		
13	12/04/2011	15/04/2011	75.5	32.4	2.33	52.9	28.4	1.86	thoracic intervation	
14	19/10/2010	07/09/2012	39.4	22.9	1.72	42.4	26.9	1.6		
15	11/04/2012	05/01/2012	42.4	31.7	1.33	36.4	25.8	1.41		
16	25/12/2009	28/12/2010	37.2	28.5	1.3	37.7	28.9	1.3		
17	01/02/2011	12/01/2011	48.9	37.7	1.29	49.6	25.8	1.92	thoracic intervation	
18	23/01/2012	20/02/2012	53.7	21.2	2.53	37.2	30.5	1.21	incorrect patient's position	
19	11/11/2011	17/05/2011	48.2	27.1	1.77	48.7	24.7	1.97	kyphoplasty	
20	19/05/2011	06/04/2011							rotation deformity	
21	07/01/2011	30/11/2010	43	33	1.3	42.3	33	1.28		
22	17/01/2012	16/02/2012	48	24.2	1.98	39.7	19.9	1.99		
23	22/05/2011	24/05/2011	50.9	27.8	1.83	40.3	23.1	1.74		
24	21/06/2011	25/06/2011	41.8	24.1	1.73	40.4	23.8	1.69		
25	26/04/2011	04/05/2011	51.8	26.8	1.93	51.6	26.6	1.93		
26	10/03/2011	12/03/2011	50.1	27.1	1.84	52.2	29.1	1.79		
27	18/01/2012	07/06/2012	52.3	29.1	1.79	44.3	26.4	1.67		
28	30/05/2011	03/06/2011	57.5	31.7	1.81	60.2	30.8	1.95		
29	16/11/2011	20/11/2011	63.5	32.4	1.95	55.6	29.8	1.86		
30	08/05/2012	11/07/2012	58.2	27.8	2.09	57.6	27.2	2.11		
31	19/08/2011	25/08/2011	60.4	28.6	2.11	56.5	26	2.17		
32	11/06/2011	18/06/2011	50.2	26.5	1.9	59.3	30.5	1.94		
33	20/03/2012	18/11/2011	42.5	20	2.125	45.6	21.8	2.09		
34	09/06/2012	26/11/2012	28.2	20.9	1.34	28	19.9	1.4		
35	03/01/2011	30/12/2010	41.3	27.1	1.52	50.5	34.6	1.46		
36	13/03/2012	25/03/2012	50.9	29.7	1.71	46.3	27.1	1.71		
37	13/01/2011	02/02/2011	37.7	27.8	1.35	39.1	26.1	1.49		
38	28/03/2011	03/03/2011	59.5	40.3	1.47	58.2	39.6	1.47		
39	16/05/2011	23/05/2011	48.9	33.7	1.45	44.8	28.3	1.58		
40	28/11/2011	13/01/2012	43	25.8	1.67	41.7	23.1	1.8		
41	17/09/2011	15/09/2011	52.2	30.4	1.72	51.5	23.1	1.59		
42	27/11/2011	08/12/2011	44.8	28.4	1.56	49.8	29.3	1.7		
43	03/05/2011	21/09/2011	38.8	25.9	1.49	41	28.4	1.44		
44	13/07/2012	01/06/2011	52.3	31.1	1.68	50.2	31.7	1.58		
45	04/11/2011	25/10/2011	43	30.4	1.41	36.3	23.8	1.52		
46	12/09/2011	13/09/2011	48.4	38.4	1.26	50.9	37.1	1.37		
47	04/04/2012	14/01/2012	43	24.5	1.75	48.7	26.6	1.83		
48	17/02/2011	16/05/2011	46.9	23.9	1.93	57.5	29.2	1.96		
49	29/10/2011	31/10/2011							incorrect patient's position	
50	18/10/2011	21/09/2011	61.5	40.3	1.53	47.7	32.4	1.47		
51	03/02/2012	31/01/2012							incorrect patient's position	
52	15/03/2012	28/02/2012							incorrect patient's position	
53	30/01/2011	19/01/2011	54.9	41	1.34	46.3	35	1.32		
54	30/05/2011	15/06/2011	48.3	21.8	2.21	52.3	22.6	2.31		
55	08/11/2011	01/12/2011							incorrect patient's position	
56	22/05/2012	27/04/2012	54.4	27.2	2	62	30	2.06		
57	28/06/2011	24/06/2011							thoracic intervation	
58	12/01/2012	16/09/2011							incorrect patient's position	
59	24/03/2011	31/03/2011							thoracic intervation	
60	03/11/2011	08/11/2011							thoracic intervation	
61	23/01/2012	05/01/2011							fracture T11	
62	05/06/2012	22/05/2012	41.4	30.8	1.34	46	33	1.39		
63	02/11/2011	22/08/2011							incorrect patient's position	
64	15/03/2011	06/03/2011	59.6	37.7	1.58	56.3	33.7	1.67		
65	18/02/2011	15/02/2011	47.1	37.8	1.25	46.9	37	1.26		
66	07/10/2011	01/12/2011	47.7	26.5	1.8	62.1	37.7	1.64		
67	16/12/2010	21/01/2011							thoracic intervation	
68	03/11/2011	14/01/2011							incorrect patient's position	
69	15/07/2012	19/06/2012								
70	07/03/2012	09/03/2012							incorrect patient's position	
